# Metabolic Alterations, Aggressive Hormone-Naïve Prostate Cancer and Cardiovascular Disease: A Complex Relationship

**DOI:** 10.3390/medicina55030062

**Published:** 2019-03-07

**Authors:** Simona Di Francesco, Iole Robuffo, Marika Caruso, Giulia Giambuzzi, Deborah Ferri, Andrea Militello, Elena Toniato

**Affiliations:** 1 Department of Medical and Oral Sciences and Biotechnologies, G. D’Annunzio University of Chieti-Pescara, 66100 Chieti, Italy; marikacaruso@hotmail.it (M.C.); giulia.giambuzzi@libero.it (G.G.); deborah.ferri@gmail.com (D.F.); e.toniato@unich.it (E.T.); 2 Department of Urological, Biomedical and Translational Sciences, Federiciana University, 87100 Cosenza, Italy; andreami65@yahoo.it; 3 Institute of Molecular Genetics, National Research Council, Section of Chieti, 66100 Chieti, Italy; ROBUFFO@unich.it; 4 Urology and Andrology Section, Villa Immacolata Hospital, 01100 Viterbo, Italy

**Keywords:** prostate cancer, obesity, diabetes mellitus, systemic arterial hypertension, dyslipidemia, cardiovascular disease

## Abstract

*Background*: Epidemiological studies suggest a possible relationship between metabolic alterations, cardiovascular disease and aggressive prostate cancer, however, no clear consensus has been reached. *Objective*: The aim of the study was to analyze the recent literature and summarize our experience on the association between metabolic disorders, aggressive hormone-naïve prostate cancer and cardiovascular disease. *Method*: We identified relevant papers by searching in electronic databases such as Scopus, Life Science Journals, and Index Medicus/Medline. Moreover, we showed our experience on the reciprocal relationship between metabolic alterations and aggressive prostate cancer, without the influence of hormone therapy, as well the role of coronary and carotid vasculopathy in advanced prostate carcinoma. *Results*: Prostate cancer cells have an altered metabolic homeostatic control linked to an increased aggressivity and cancer mortality. The absence of discrimination of risk factors as obesity, systemic arterial hypertension, diabetes mellitus, dyslipidemia and inaccurate selection of vascular diseases as coronary and carotid damage at initial diagnosis of prostate cancer could explain the opposite results in the literature. Systemic inflammation and oxidative stress associated with metabolic alterations and cardiovascular disease can also contribute to prostate cancer progression and increased tumor aggressivity. *Conclusions*: Metabolic alterations and cardiovascular disease influence aggressive and metastatic prostate cancer. Therefore, a careful evaluation of obesity, diabetes mellitus, dyslipidemia, systemic arterial hypertension, together with a careful evaluation of cardiovascular status, in particular coronary and carotid vascular disease, should be carried out after an initial diagnosis of prostatic carcinoma.

## 1. Introduction

Androgens play a key role in the development, growth and maintenance of prostate cells, as well as carcinogenesis and prostate cancer (PCa) progression [1,2]. Androgen dependence of PCa was first demonstrated in humans by Huggins in 1941 [3]. This assumption rationalized the “Historical myth” of androgen deprivation therapy for decades as a gold standard treatment in advanced and metastatic PCa [4,5]. After an average time of 12 to 33 months, despite the testosterone castration levels, PCa recovered and evolved towards a castration-resistant stage with limited effective treatment options [6]. Median survival of castration-resistant metastatic PCa was estimated not to exceed 25 months [7].

Metabolic alterations play a key role in manifesting this mechanism. Particularly PCa express profound metabolic reprogramming favoring biosynthesis processes and limiting catalytic mechanisms. Therefore, the metabolism of PCa represents a new therapeutic target and can offer new opportunities in the prevention and diagnosis of advanced PCa, independently from androgen ablation therapy [8,9,10,11].

Although numerous studies showed a possible association between metabolic disorders and aggressive PCa, the literature has often been limited and unambiguous.

Given that more and more epidemiological investigations showed that most patients with PCa died due to causes other than cancer, especially for cardiovascular events [12,13,14], the aim of the study was to analyze the recent literature and summarize our experience on the association between metabolic alterations and aggressive hormone-naïve PCa as well as the prevalence of cardiovascular damage, both at the initial diagnosis and in the advanced metastatic stage.

## 2. Materials and Methods

### 2.1. Identification of Studies

We identified significant recent papers in electronic databases as Scopus, Life Science Journals, and Index Medicus/Medline.

Studies were discovered using the following key words: prostate cancer, diabetes mellitus, dyslipidemia, obesity, systemic arterial hypertension, cardiovascular disease.

### 2.2. Inclusion Criteria

Working separately, reviewers selected all qualified studies in full text. To be included, articles had to (1) evaluate the association between aggressive PCa, metabolic alterations and/or cardiovascular disease; (2) contain an original data analysis and (3) from a peer reviewed journal. Articles were excluded if the clinical study (1) did not analyze a reciprocal relationship between PCa, metabolic alterations and/or cardiovascular disease (2) presented only as a case report or had an inappropriate design.

### 2.3. Our Experience

In the second part of article we finally focused and synthesized our personal experience regarding the relationship between aggressive hormone-naive PCa, metabolic disorders and cardiovascular damage.

## 3. Results and Discussion

### 3.1. Literature Review

#### 3.1.1. Prostate Cancer and Diabetes Mellitus

The conversion of glucose to ATP, even in the presence of high levels of oxygen, occurs via the anaerobic effect (Warburg effect) in tumor cells due to the irreversible damage of the respiratory chain in the first phase of cancerogenesis. This results in ATP production at a rate 100 times faster than mitochondrial oxidation [8]. In the early stages of PCa, a widespread increase in lipogenesis directly coupled with glucose and glutamine metabolism is associated with PCa progression, worse prognosis and shorter survival [15,16].

Epidemiological studies show a conflicting association between Type 2 diabetes mellitus (DMII) and PCa. These inconsistent results are attributed to changes in insulin concentration during DMII development and progression. The risk of PCa, in particular, is associated with high insulin levels and insulin resistance. Consequently, it has hypothesized that the incidence rate of PCa increased in subjects with recent diagnosis of DMII, characterized by hyperglycemia and hyperinsulinaemia, and decreased with DMII progression [17,18,19]. Hyperinsulinaemia appears to be linked not only to a higher incidence of PCa, but also to an increased Gleason Score (GS) and cancer mortality, although not all studies are consistent [20,21,22,23,24,25,26].

The protect study showed that DMII was inversely associated only with differentiated PCa (Gleason Score 2–6), but not with poorly differentiated PCa [27]. Other studies, however, showed an inverse association between DMII and PCa for both high and low-grade tumors [22,28,29].

In the investigations in which an inverse relationship between DMII and PCa has been observed, this association appears to be more significant with the duration of DMII, but this does not seem to conform with all the studies that have examined the duration [28].The unanimity of the results was negatively affected by the absence of discrimination in diabetic status in clinical trials and mostly by inaccurate selection of comorbidities. This reflected a different impact of DMII on the incidence of PCa and in particular on tumor aggressivity [17,23,30,31,32]. Moreover, recent studies suggest that obesity, hypertension, DMII, hypercholesterolemia, in combination, can play a role in the development of high-grade PCa (GS ≥ 7) as well as in tumor progression [16,33]. Western diet with a high carbohydrate rate supports tumor progression in murine models and a high protein diet with a low-carbohydrate diet inhibits intratumoral androgen synthesis and castration-resistant cancer growth [34]. Therefore, it’s possible to hypothesize how a Mediterranean regimen characterized by a high consumption of vegetables, fruits, legumes, nuts, fish, can positively impact on a natural history of PCa and concomitant cardiovascular disease [35,36,37,38].

Moreover diet/genome interactions may influence the metabolic response to diet components and the susceptibility to PCa risk and progression, directly or indirectly altering gene expression or structure, while also modulating cell proliferation/apoptosis, DNA damage and repair, inflammation, oxidant/antioxidant balance and angiogenesis. Consequently, dietary interventions based on nutritional status, genotype analysis and “precision nutrition” can be used to establish specific nutritional guidelines to prevent or cure metabolic alterations and PCa [39,40,41].

#### 3.1.2. Prostate Cancer and Dyslipidemia

Prostate cancer cells have an altered metabolic homeostatic control and increased endogenous cholesterol biosynthesis, with accumulation in tumor cells [42]. Cholesterol plays a key role in metastatic tumor progression by acting as a mediator in cell proliferation, inflammation and steroidogenesis [43,44].

Epidemiological and pre-clinical studies suggest that elevated levels of serum cholesterol could play a role in PCa progression through increased production of androgens by prostate cancer cells and activation of androgen receptors [42,45,46].

**Figure 1:**
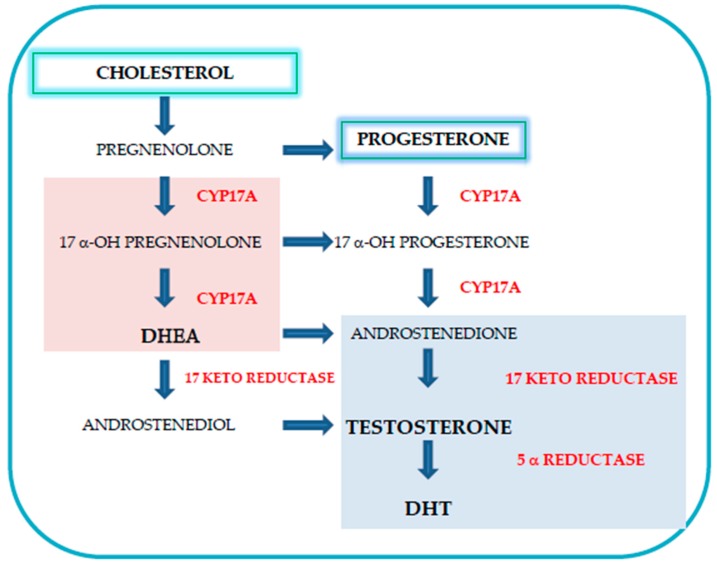
Intratumoral de novo steroid synthesis from circulating serum cholesterol. Increased cholesterol and progesterone levels have been identified in prostate cancer along with an increased expression of enzymes involved in steroidogenesis, in particular, CYP17A, 17-keto-reductase and 5α-reductase 1.

Cholesterol would then act as the precursor of intratumoral androgen biosynthesis [42,47]. Increased serum cholesterol levels were correlated with tumor volume, intratumoral testosterone levels and the expression of key steroid genes, such as CYP17A, suggesting that one of the intratumoral androgen sources would be represented by de novo steroid synthesis from circulating serum cholesterol [42]. Tumors in hypercholesterolemic environment also exhibits lower levels of apoptosis, increased activation of Akt kinase, related to aggressive PCa and increased vascularization [48,49]. Animal pre-clinical studies demonstrate that hypercholesterolemia was associated with increased PCa risk and metastatic progression [50]. In addition, increased cholesterol levels have been identified in PCa bone metastases, along with an increased expression of enzymes involved in steroidogenesis. In particular, the effects of CYP17A (which converts progestins into androgens), 17-keto-reductase (which converts androstenedione into testosterone) and 5α-reductase 1 (which converts testosterone into dihydrotestosterone) [51,52,53] can be seen in Figure 1.

Overall clinical trials reveal that hypercholesterolemia causes increased risk of PCa or aggressive disease, while statins reduce the risk of advanced disease [43,54,55,56].The mevalonate pathway was essential for the production of cholesterol and isoprenoids and had direct effects on the growth and progression of prostate cancer cells [57,58].The transcriptional regulation of the enzymes involved in this pathway occurs through the activation of SREBP-2, a regulator of androgenic synthesis which is itself regulated by androgens [59].The SREBP-2 expression increased during tumor progression, was significantly higher after castration and lost its inhibition feedback in tumor cells [55]. The drugs that most inhibited the Mevalonate pathway are nitrogen-containing bisphosphonates (N-BPs) and statins. Both have antitumor activity through effects on both bone tissue and cholesterol metabolism. In particular, observational studies show an inverse association between the use of statins and PCa progression, with a significant reduction in the risk of advanced disease with long-term use of statins, especially when used for more than 5 years and before tumor development [47,60,61,62,63,64]. These data suggest that an early approach based on the reduction of cholesterol levels before the onset of PCa could be an effective prevention and treatment strategy for PCa. In particular, prostate cancer cells increased the expression of Farnesil Pyrophosphate Synthetic Enzyme (FPPS), a key enzyme of the mevalonate pathway, detecting its correlation with PCa progression [65]. This enzyme is an important mediator of T cell activity essential for the elimination of tumor cells through the immune system [66]. FASN (Fatty Acid Synthase) catalyzed the synthesis of palmitate through the condensation of malonyl-CoA and acetyl-CoA. Its expression progressively increased during the natural history of PCa and reached maximum values in the androgen-independent PCa with bone metastases [67]. FASN inhibitors have been used to dysregulate lipid metabolism inhibiting PCa progression and causing cell death. FASN inhibition showed citotoxic and anti-proliferative activity in PCa cells, inducing apoptosis and reducing the lipid content in cancer cells in a concentration and time-dependent manner. FASN inhibitors induced degeneration and atrophy of prostatic tissue independently of the androgen status [68,69].

#### 3.1.3. Prostate Cancer and Obesity

Adipose tissue is an active endocrine tissue capable of regulating prostate metabolic activity and influencing carcinogenesis and especially tumor progression [70,71,72,73,74,75].

The relationship between PCa and obesity, although subject to numerous studies, remains controversial at present. A possible explanation for this is due to the insufficient evaluation and control of possible concomitant metabolic factors associated with obesity such as systemic arterial hypertension (SAH), DMII, and dyslipidaemia, as well as a different design of the studies. Recent studies showed that the association between PCa and obesity could vary according to tumor grade, highlighting a direct relationship between adipose tissue and aggressive cancer and an inverse association with low-grade tumors [76,77,78,79,80,81]. However, not all studies were consistent and some clinical trials showed no relationship between obesity and tumor aggressiveness [82,83,84].

Leptin and adiponectin, adipokines produced by adipose tissue, regulated the action of Insulin on the glucose cellular uptake and reduced the metabolism of fatty acids. Leptin increased lipolysis, insulin sensitivity, inflammation and thromboembolism. In obesity in particular, leptin was increased and negatively affected cell differentiation, the risk of tumor progression, cell migration and prostate angiogenesis, by inducing pro-angiogenic factors [78,79,80,81,82,83,84,85,86].

Adiponectin, on the other hand, was reduced in obesity and adversely affected the histological grade and stage of PCa. In vitro studies showed that Adiponectin inhibited cell growth and proliferation in PCa and antagonized the proliferative effects of Leptin and IGF-1 in the androgen-independent PCa [87]. In addition to its effects on prostate cell proliferation, adipose tissue reduced the production of anti-inflammatory adipocytes such as adiponectin and increased the release of pro-inflammatory adipocytes such as leptin causing a chronic inflammatory state and prostatic tumor progression [6,74,78]. In particular, the expansion of adipose tissue caused the development of an inflammatory environment with increased secretion of cytokines such as IL-6, TNFα and MCP1. They in turn acted as attractants for further immune cells and created an environment that perpetuates immune infiltration and the production of inflammatory cytokines. Lipid inflammatory mediators such as arachidonic acid, eicosanoids, prostanoids and leukotrienes had also been increased [88]. Hormonal and inflammatory obesity-related alterations could therefore significantly contributed to prostate tumor growth and progression through the promotion of mitogenesis (Leptin, IGF-1, Insulin etc), angiogenesis (VEGF, IL6, IL8 etc) and tumor invasiveness (Leptin, IL6, etc.) [47,89].

#### 3.1.4. Prostate Cancer and Systemic Arterial Hypertension

Only few studies have explored the association between SAH and PCa. Beebe-Dimmer et al. found a positive association between SAH and PCa [90]. Han et al. showed that diastolic pressure was positively associated with Prostate Specific Antigen (PSA) serum levels [91]. Subsequent studies, however, did not demonstrate a statistically significant association between SAH and PCa incidence [16]. On the other hand, a significant relationship between arterial SAH, advanced PCa and the risk of biochemical recurrence was demonstrated [92,93,94,95]. It has also been showed that patients with PCa with SAH and overweight had a significantly lower survival time than control subjects [94]. Preclinical evidence showed that the use of Beta Blockers could affect PCa progression; in particular, sympathetic nerve stimulation was able to induce metastases in prostate tumor models and the administration of beta blockers was capable to prevent the effect of adrenergic stimulation on the promotion of metastases [96,97]. The main mediator of these effects was the β-adrenergic receptor, which was also implicated in the tumor-immune system response [98]. Moreover, recent clinical trials confirmed these findings in humans by demonstrating that the use of Beta Blockers was associated with reduced mortality in patients with high-risk or metastatic PCa at the time of diagnosis [99,100].

### 3.2. Personal Experience

#### 3.2.1. Obesity and Aggressive Prostate Cancer

In our experience, similar to the most recent literature, we showed that obesity was associated, both individually and in combination with cardiovascular risk factors, with aggressive PCa. A reverse association was instead observed between obesity and non-aggressive PCa, at initial diagnosis [101]. In particular, obese patients with early PCa had a higher risk of developing high-grade PCa compared to non-obese patients with PCa [101,102,103]. Several hypotheses could explain the association between obesity and high-grade PCa. The literature suggested that obesity could be associated with biological changes (e.g., inflammation, insulin resistance, angiogenesis, cell migration) and modification of adipokine levels related to aggressive tumor phenotype. It had also been reported that Adiponectin was involved in the regulation of metabolic homeostasis (in particular regulation of glucidic and lipid metabolism), inhibition of inflammation, atherogenesis, angiogenesis and cell migration [104,105,106].

Adiponectin levels were reduced in obesity, coronary heart disease and tumors such as PCa, with a negative association with histology and cancer stage [76,107]. In vitro, adiponectin inhibited cell growth and proliferation in the prostate, while in androgen-independent cancer tumor cells antagonized the proliferative effects of leptin and IGF-1 [85].

Leptin act directly on prostate cancer cells by affecting steroid activity, cell cycle regulation, and insulin activity [108]. In particular, Leptin increased lipolysis, insulin sensitivity, inflammation and thromboembolism [104]. It influenced cellular differentiation and cancer progression, cell migration, tumor angiogenesis and promoted advanced PCa through induction and activation of pro-angiogenic factors [78,85,86,109,110].

It was hypothesized that low adiponectin levels and high levels of leptin and resistin observed in obese patients could be involved in prostate cancer aggressiveness. Recent studies confirmed these hypotheses, showing that high levels of leptin could be correlated with a worse grade and tumor stage, while high levels of adiponectin were associated with a less aggressive tumor grade and stage [78,81,111]. This suggests that low levels of adiponectin and high levels of leptin observed in obese patients could be related with the development of high-grade PCa.

In vitro studies confirmed these results showing that specific genetic polymorphisms in leptin and adiponectin as their receptors were associated with PCa proliferation and progression, through increased inflammation, angiogenesis, worse pathological grade, metabolic syndrome, closely associated with aggressive PCa [111,112,113].

#### 3.2.2. Obesity, Diabetes Mellitus and Aggressive Prostate Cancer

Obesity was a well-known risk factor for DMII. Since obesity was a factor involved both in the development of DMII and in the aggressive PCa, we analyzed the possibility of an association between DM and aggressive PCa hormone-naïve and demonstrated that DMII was associated with high-grade PCa, but only in obese subjects [101]. These observations agreed with the data reported in recent studies and suggested that DMII could be associated with aggressive PCa, but only in patients with concomitant obesity, whereas DMII wasn’t associated with high-grade PCa in non-obese patients [23,31,114]. Recent data from 119315 subjects with DM in order to examine the relationship between Metformin exposure and PCa risk indicate that there is no association between the use of Metformin and PCa respect to tumor grade [115]. However, the effects of antidiabetic agents in PCa development, aggressiveness and progression remain unclear. Specifically, the literature reports a conflicting relationship between the use of Metformin and PCa, demonstrating a reduced or increased risk or no association [116,117,118,119,120,121,122,123]. It is possible to hypothesize that a combination of biological factors may be responsible for the discrepant association between DMII and high-grade PCa. The link between obesity, DMII and PCa may be related to insulin resistance, hyperinsulinemia, reduced IGFBP (Insulin-like Growth Factor Binding Proteins), increased bioavailability of IGF-1 (Insulin-like Growth Factor-1), steroid hormones (reduced levels of androgens, increased estrogen Levels) and inflammatory markers [124,125,126]. In particular, hyperinsulinemia promoted prostatic cancer aggressiveness and progression [127]. In addition, insulin secretory capacity was higher in obese patients with DMII than non-obese patients with DMII [125]. DMII and obesity were associated with low serum testosterone levels and increased estrogen levels, correlated with high-grade PCa [23]. Some reports have suggested that low levels of testosterone were associated with aggressive PCa, inadequate treatment after radical prostatectomy, high-grade PCa (Gleason Score 8–10) and locally advanced prostate cancer (pT3 and pT4) [128,129,130,131]. These observations suggest a possible role of sex steroid hormones in the contribution of obesity and DMII in high-grade PCa.

Systemic inflammation can also contribute to increased tumor aggressivity in obesity and DMII. DMII and obesity are in fact inflammatory conditions associated with increased cytokine production such as Tumor necrosis factor (TNFα), Interleukin (IL) 6 and IL8 [132]. These cytokines stimulate the Nuclear Factor KappaB pathway (NF-kB), directly related to lymph node invasion and androgen-independent progression in PCa [133,134]. We have previously reported an important role of inflammation and the immune system as regulators of physiology and pathology of PCa [101,134]. Chronic inflammation and oxidative stress associated with both DMII and obesity could thus contribute to PCa development and progression [135,136].

#### 3.2.3. Systemic Arterial Hypertension, Dyslipidemia and Aggressive Prostate Cancer

We analyzed the association between arterial hypertension and PCa in patients with PCa hormone naïve both at the initial diagnosis [103] and in the advanced metastatic phase [137]. We showed that the presence of arterial hypertension and obesity were significantly associated with aggressive PCa (OR 2.84, p < 0.05). In non-obese patients, however, no relationship was established between high blood pressure and PCa.

Previous studies have analyzed the association between arterial hypertension and PCa, but the results were conflicting, demonstrating either reduced risk or a positive association [93,138,139,140]. Similarly, the use of antihypertensive drugs as beta-blockers was linked to both decreased risk of PCa and cancer mortality as well as to an increased risk of PCa progression and death, showing inconclusive results [100,141,142,143].

**Figure 2:**
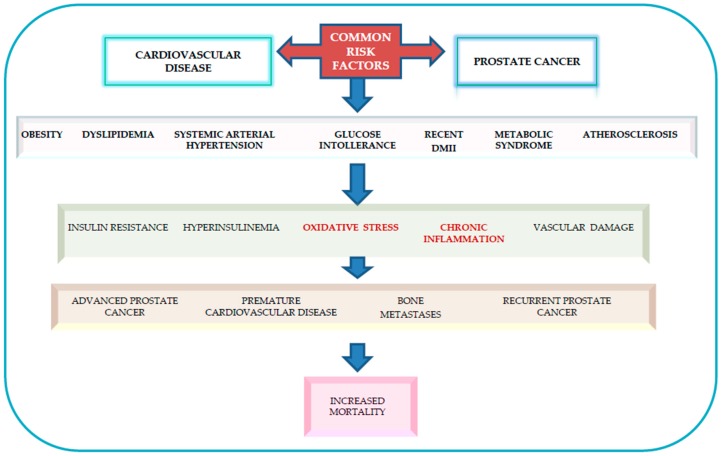
Reciprocal relationship between prostate cancer and cardiovascular disease. Common risk factors link prostate cancer and cardiovascular disease. It is possible to hypothesize that this relation has a direct association with common metabolic components as obesity, dyslipidaemia, systemic arterial hypertension, glucose intolerance, recent type II diabetes mellitus, metabolic syndrome, atherosclerosis. These factors are associated with Insulin Resistance, hyperinsulinemia, oxidative stress, inflammation and precocious vascular damage and are related to advanced prostate cancer, bone metastases, biochemical recurrence and increased tumor mortality.

Pathogenetic mechanisms that potentially link obesity and hypertension to PCa aggressivity are not known. It is possible to hypothesize that the link between obesity, arterial hypertension and aggressive PCa has a close relationship with common biological mechanisms such as insulin resistance, hyperinsulinemia and inflammation [144]. Chronic inflammation, reactive oxygen species and the oxidative stress associated with both hypertension and obesity could contribute to tumor progression [145,146,147]. In our experience, a statistically significant association between arterial hypertension and advanced metastatic PCa was demonstrated with an OR of 4.5. In the same study we also found that hypercholesterolemia, particularly high levels of total cholesterol and LDL-C, were significantly associated with aggressive metastatic PCa with an OR of 3.28 [137]. Recent studies indicated that hypertension, hypercholesterolemia, atherosclerosis, and a composite score of metabolic factors were associated with advanced PCa, biochemical recurrence and increased tumor mortality [28,93,139,148,149,150,151], as shown in Figure 2.

In our experience and consistent with these studies, we first highlighted a significant association between arterial hypertension (in particular systolic arterial hypertension), hypercholesterolaemia and coronary and carotid vascular disease in patients with advanced PCa and concomitant bone metastases [136].

**Figure 3:**
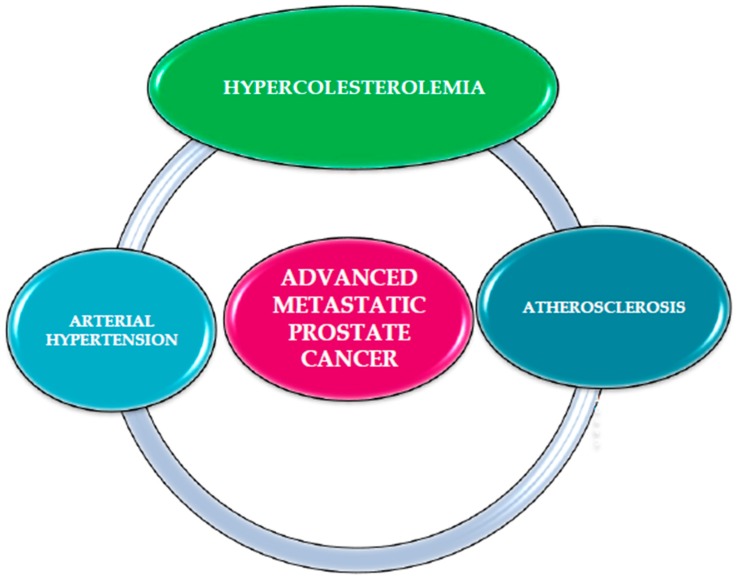
“New Players” in advanced metastatic PCa. Hypertension, hypercholesterolemia and atherosclerosis could be considered common etiopathogenetic mechanisms in advanced metastatic prostate cancer, particularly in presence of bone metastases.

If confirmed in larger studies, our research suggests that hypertension, hypercholesterolemia and atherosclerosis could be considered as “New Players” in advanced metastatic PCa and confirm common etiopathogenetic mechanisms, as shown in Figure 3. A study by Thysel et al., analyzed metabolites associated with PCa metastases and in particular identified high levels of cholesterol in bone metastases. The authors proposed that prostate cancer cells synthesize cholesterol de novo as well as the involvement of this metabolite through the surrounding environment [152], as shown in Figure 1.

#### 3.2.4. Metabolic Syndrome and Aggressive Prostate Cancer

The metabolic syndrome (MetS) is characterized by a cluster of comorbidities as visceral obesity, hyperglycemia, systemic arterial hypertension and dyslipidaemia. Epidemiological Studies showed conflicting results on the association between MetS and aggressive PCa [153,154,155,156].

Overall a lower survival time, shorter time to castration-resistant Pca, increased prostate cancer-specific mortality, and biochemical recurrence were related to MetS [153,157].

Our group firstly investigate the association between MetS and high-grade PCa without the influence of endocrine therapy, showing a significant relationship between MetS and aggressive PCa at initial diagnosis (OR 1.87, p < 0.05) and a reduced risk of low-grade PCa (OR 0.53, p < 0.05). Moreover, fasting glucose, triglycerides, waist circumference, systemic arterial hypertension were significantly associated with high-grade PCa [158].

The metabolic alterations, chronic inflammation, oxidative stress associated to MetS may contribute to PCa progression causing a chronic inflammatory condition that predisposes to aggressive and metastatic PCa [145,146,147].

#### 3.2.5. Hormone-naive Prostate Cancer and Vascular Damage

Epidemiological studies showed that cardiovascular disease is one of the major causes of death in patients with PCa (30%) [159,160,161]. In our experience we analyzed the relationship between PCa hormone-naive and carotid and coronary vascular disease, both at initial diagnosis of PCa [102,162] and metastatic cancer [136]. In particular, we showed a significant association between bone metastases and vascular pathology in advanced metastatic PCa with an OR of 3.8.

Consistent with our results, it was shown by Zoller et al. that the risk of coronary artery disease augmented in the first 6 months after the diagnosis of cancer and in the specific case of PCa this risk remained high for over 10 years. Furthermore, the risk of coronary artery disease was correlated with the presence of metastases and cardiovascular risk factors [163]. Moreover, we found that 23% of patients with hormone naïve localized PCa presented carotid vasculopathy (OR 2.43, p < 0.05), whereas 17% of patients had a history of symptomatic coronary artery disease (OR 1.88, P < 0.05). However, a limited number of studies explored the relationship between atherosclerotic vascular disease and PCa. Some studies showed no association between atherosclerotic disease and PCa; while others, similarly to our results, documented a possible relationship [164,165,166,167]. Consistent with our research in a large prospective study, a high prevalence of cardiovascular risk factors and cardiovascular disease (27%) has been demonstrated in PCa before ADT has begun. In particular, 19% and 8% of patients had cardiac ischemic disease and cerebrovascular disease, respectively [168]. Similarly, in the study REDUCES, the presence of coronary artery disease was associated with an increased risk of PCa by 35% [169]. Similar results were obtained from Stamatiou et al. in an autopsy study, as evidenced by the fact that patients with PCa had a greater risk of developing advanced coronary artery disease than those without PCa [166]. Although there are no other studies that have assessed the presence of carotid vascular damage in patients with PCa hormone naïve at initial diagnosis, the results of our research are also consistent with a prospective study of Pereira et al. which showed that myointimal carotid thickness was high at baseline in patients with PCa. In addition, another endothelial damage marker, represented by VCAM-1 levels, was reduced in PCa patients after radiotherapy.

The authors hypothesize that the inflammatory process associated with neoplasia may have been reduced with the treatment, despite the potential adverse effects of irradiation on vascularization.

It has been widely accepted in international literature that cancer cells can cause inflammatory conditions characterized by high levels of inflammatory biomarkers such as reactive protein C, prostaglandins, adhesion molecules, etc. [134,170,171]. If confirmed in other studies, the results of our research suggest that CAD and CVD may represent new risk factors in PCa and suggest common etiopathogenetic factors.

Atherosclerotic cardiovascular pathology and PCa could share common etiopathogenetic mechanisms. Therefore, we hypothesized that they could be present in patients with PCa from initial cancer diagnosis even in the absence of hormone therapy [162] and could be associated with subsequent phases of PCa progression to a more aggressive and metastatic phenotype [102,137]. Since inflammation was a factor that accompanies both atherosclerosis and activation of hemostasis and tumor pathology, a possible relationship between inflammation, PCa, hemostatic activation and vascular damage has been hypothesized. Based on this hypothesis, therefore, effective tumor treatment could reduce tumor volume, associated inflammation, hemostatic activation and hence the risk of vasculopathy [163].

## 4. Conclusions

Recent literature and our research showed that metabolic alterations and coronary and carotid vascular disease influenced aggressive and metastatic PCa. Therefore, a careful evaluation of cardiovascular risk factors should be carried out from the initial diagnosis of PCa, in particular focusing on risk factors such as obesity, arterial hypertension and dyslipidaemia, together with a careful evaluation of the concomitant presence of cardiovascular disease.

Overall, the results of our research add further motivation to control metabolic factors in PCa from initial cancer diagnosis, even in the absence of hormone therapy, in order to reduce cardiovascular risk and tumor progression. In addition, our results suggest that increased oxidative stress and a permanent inflammatory state may predispose to a more aggressive and metastatic tumor phenotype. Metabolic alterations can in fact create a favorable environment for tumor progression and cause an inflammatory state that predisposes to vascular disease and PCa.

On the other hand, chronic inflammation and oxidative stress associated with metabolic damage and atherosclerosis may also contribute to tumor progression, causing premature vascular and prostatic damage.

The metabolic and cardiovascular profile evaluation at initial diagnosis of PCa, particularly dyslipidemia, glucose intolerance, obesity, metabolic syndrome, might be used to discover new markers for PCa monitoring and more importantly to identify novel personalized integrative therapies targeting critical metabolic changes that sustain proliferation, migration, and invasion of PCa cells.
